# Peer Review of “Machine Learning and Medication Adherence: Scoping Review”

**DOI:** 10.2196/33965

**Published:** 2021-11-24

**Authors:** Przemyslaw Kardas

**Affiliations:** 1 Department of Family Medicine Medical University of Lodz Lodz Poland

*This is a peer-review report submitted for the paper “Machine Learning and Medication Adherence: Scoping Review”.*

## Round 1 Review

### General Comments

This paper [1] covers a very interesting area on the use of machine learning for assessment of medication adherence, yet in its current version, it does not add a lot to the field. It is a pity, as it seems that the authors performed their review well. However, the presentation of the results is not acceptable.

### Major Comments

1. It creates a lot of confusion that the authors use “adherence” instead of “compliance.” In fact, these two are equivalent terms, of which adherence is preferred and compliance is a bit old-fashioned. The authors need to define the major concept they use, and these two need to be carefully checked against available literature and the ABC taxonomy.
2. The Abstract provides no numeric data; even the number of identified publications is missing. Similarly, the conclusions of the Abstract are inconclusive.
3. The authors mentioned previous reviews in this area, yet they did not make it clear what was different about their own work. What exactly was missing in the previous reviews that turned them toward this new exercise?
4. Publication selection for review: What were the criteria used to identify acceptable papers in the full-text review? What was the reason for screening a sample of 20 papers first?
5. “Medication adherence activities” is not a term used in the literature to describe interventions aimed at assessment or modification of medication adherence. Please use another term that is used in the existing literature.
6. The paper is lacking a lot of details; for example, what was the basis for the dichotomization of the source databases into “biomedical” and “computer” in Figure 3?
7. In Tables 1-3, instead of simply providing the number of the reference, it is also advisable to have the first author’s name and the year of the publication.
8. I have a feeling that the studies listed in Table 1, based on self-report and pharmacy claims data, do not “predict” adherence but rather assess it. Please correct me if I am wrong.
9. The paper must be self-explanatory; therefore, abbreviations such as DOT need to be explained. When addressing a general audience, it makes sense to do the same with the abbreviations of algorithms cited within.
10. Numbers, numbers, numbers, please! The Results section reads, for example, “LEAP had the best prediction accuracy of the machine learning methods used”—by how much? Was the difference statistically significant?
11. Being a clinician, I feel that this might be information technology (IT) jargon: “The first of these articles used data collected during hospital stays to generate features” (from Results). However, please make sure that the text is also meaningful for non-IT people.
12. In light of previous publications in the field, the first sentence of the Discussion needs to be rechecked.
13. In the Discussion, the authors say “However, more work needs to be done to better understand the impact of socioeconomic status [on adherence].” In fact, a lot of work has been done in that area, and it would help the paper if authors would broaden their understanding of it.
14. From the Discussion: “Some of these works compared the different types of algorithms to determine which was the most accurate...” Which ones? Please cite!
15. To conclude, it needs to be stressed that the authors should extract a lot more data and conclusions from the material they reviewed—instead of saying “some studies...,” please provide the numbers (eg, “over 40% of studies found the parameter to change by >90%”).

## Round 2 Review

### General Comments

This version of the manuscript is a lot more advanced than the previous one yet still far from the target. Because of the importance and novelty of the topic, it still makes sense to work on making the paper better. Below are my suggestions.

NOTE: I have activated the line numbering in the original manuscript to make my remarks more precise. To make sure that we use the same numbering, the line with the “ABSTRACT” heading was numbered 18.

### Major Comments

1. In the body of text, you refer to the help and advice provided by two librarians and two pharmacists, yet it seems to me that they are not included in the authorship nor thanked in the Acknowledgments. Please take care of solving this.
2. Overall, the interesting work done in this exercise is not followed with a clear description. In fact, it is very hard to learn what exactly the use of machine learning was in the context of medication adherence or the outcomes of this process. These, however, were the major objectives of this paper. In such a case, the conclusion from the Abstract stating that “Machine learning has the potential to greatly improve medication adherence” seems to be unsupported by the data presented.

### Additional Suggestions

- Line 23: The number of identified studies belongs in the Results.
- Line 26 onward: “Verb” is an uncommonly used term in this context; please search the literature to find a more frequently used equivalent.
- Line 29 onward: Using percentiles makes sense when the total number is ≥100; in this case, the number of identified publications was only 43; what justifies fractions and not the percentiles?
- Lines 42-3: The Discussion is missing in the Abstract (what is provided now is not a real discussion of the findings).
- Lines 92-3: The eligibility criteria need to be more detailed; it is unclear now what sort of relationship had to link the included publications with medication adherence, and what was the exclusion criteria?
- Line 134 refers to “predictors”—predictors of what?
- Line 136-7: What do you mean by “The data collected for this study was qualitative and sometimes quantitative”? What does “sometimes” mean in this context?
- Line 165 refers to “13 studies,” yet Figure 3 shows only 12 items in that category.
- Tables 1-3 need serious improvement. Putting all the comments together in columns placed to the right makes no sense. No idea why “Some entries were excluded for brevity,” especially in cases of short algorithm acronyms. The footnote marked ** is not applicable to Table 2.
- Table 1: I would love to see one more column describing what sort of adherence measure the machine learning algorithm was able to predict (eg, “filling the prescription” or “daily drug intake”).
- Table 1: How did you identify the “strong predictors”? Has any statistical threshold been applied to this selection?
- Table 2: I would love to see one more column describing what sort of adherence measure the machine learning algorithm was able to identify. For example, there are plenty of studies using smart pill bottles—so what exactly was the role of machine learning in [2] for it to be included in this review and not to include other studies?
- Table 3: Same as above, plus which aspect of adherence was improved—the one that was tested; the other one?
- Line 210, 213: Correct “99 DOTS” to “99DOTS.”
- Line 221, 222: “The next paper used face recognition software and computer vision to monitor medication adherence”—which aspect of medication adherence are you considering here?
- Line 241-2: “These assessments were then used to create predictors”—predictors of what? I guess not of medication adherence, if you say that medication adherence was a...predictor!
- Line 247-50: Usually, limitations are provided at the end of the Discussion.
- Line 285-6 states: “Approximately 87% of these studies used either logistic regression, artificial neural networks, support vector machines, or random forest algorithms.” Why is this not visible in Table 1?
- Lines 282 and 342 still use the term “compliance” instead of “adherence.”
- Lines 288-291: You provide comparisons of the accuracy of diverse algorithms yet without any statistical significance values. That sort of simple comparison is not inconclusive

## Abbreviations

IT: information technology
